# Yeast lipids from cardoon stalks, stranded driftwood and olive tree pruning residues as possible extra sources of oils for producing biofuels and biochemicals

**DOI:** 10.1186/s13068-018-1142-8

**Published:** 2018-05-23

**Authors:** Giorgia Tasselli, Sara Filippucci, Elisabetta Borsella, Silvia D’Antonio, Mattia Gelosia, Gianluca Cavalaglio, Benedetta Turchetti, Ciro Sannino, Andrea Onofri, Silvio Mastrolitti, Isabella De Bari, Franco Cotana, Pietro Buzzini

**Affiliations:** 1 Department of Agricultural, Food and Environmental Sciences, Industrial Yeasts Collection DBVPG, University of Perugia, Perugia, Italy; 2 CIRIAF-Biomass Research Centre, University of Perugia, Perugia, Italy; 3 ENEA-Italian National Agency for New Technologies, Energy and Sustainable Economic Development, Matera, Italy; 4 Department of Engineering, University of Perugia, Perugia, Italy

**Keywords:** Mediterranean lignocellulosic biomass, *Solicoccozyma terricola*, Lipids, Fatty acid profiles

## Abstract

**Background:**

Some lignocellulosic biomass feedstocks occur in Mediterranean Countries. They are still largely unexploited and cause considerable problems due to the lack of cost-effective harvesting, storage and disposal technologies. Recent studies found that some basidiomycetous yeasts are able to accumulate high amount of intracellular lipids for biorefinery processes (i.e., biofuels and biochemicals). Accordingly, the above biomass feedstocks could be used as carbon sources (after their pre-treatment and hydrolysis) for lipid accumulation by oleaginous yeasts.

**Results:**

Cardoon stalks, stranded driftwood and olive tree pruning residues were pre-treated with steam-explosion and enzymatic hydrolysis for releasing free mono- and oligosaccharides. Lipid accumulation tests were performed at two temperatures (20 and 25 °C) using *Leucosporidium creatinivorum* DBVPG 4794, *Naganishia adeliensis* DBVPG 5195 and *Solicoccozyma terricola* DBVPG 5870. *S. terricola* grown on cardoon stalks at 20 °C exhibited the highest lipid production (13.20 g/l), a lipid yield (28.95%) close to the maximum theoretical value and a lipid composition similar to that found in palm oil. On the contrary, *N. adeliensis* grown on stranded driftwood and olive tree pruning residues exhibited a lipid composition similar to those of olive and almonds oils. A predictive evaluation of the physical properties of the potential biodiesel obtainable by lipids produced by tested yeast strains has been reported and discussed.

**Conclusions:**

Lipids produced by some basidiomycetous yeasts grown on Mediterranean lignocellulosic biomass feedstocks could be used as supplementary sources of oils for producing biofuels and biochemicals.

## Background

The use of some non-food oilseed crops as sources of industrially important oleochemicals is generally considered a great opportunity for reducing human dependence from fossil oils and for creating a sustainable “green” industry [1]. Depending on their fatty acid (FA) profiles, triacylglycerols (TAGs) from oilseed crops can be converted into biofuels and building blocks for lubricants, adhesives, solvents, biosurfactants, cosmetics, and degradable polymers [2]. However, the cultivation of oilseed crops may reduce the surface of agricultural soil normally destined to food crops, thus causing a possible economic impact on food prices, also in view of the increasing nutritional demand of world human population [3]. In this context, microbial oils can be considered a supplementary source of lipids for meeting the needs of a growing market of bio-based oleochemicals [4].

Many studies reported that the lipid production of oleaginous yeasts (and their FA profile) depends on several factors, such as temperature, oxygenation, carbon and nitrogen sources, and C/N ratio [5, 6]. Under conditions of nitrogen depletion, the flow of carbon in oleaginous yeasts is converted into acetyl-CoA (via citric acid) that stimulates the synthesis and accumulation of high amounts of intracellular lipids stored in cytoplasm lipid droplets [7, 8]. Recombinant strains of *Yarrowia lipolytica* have been extensively studied for obtaining high lipid productions [9–11]. However, some recent studies found that some basidiomycetous yeasts can be considered as possible alternatives to conventional oleaginous yeasts due to their high aptitude to accumulate lipids [8, 12, 13].

Lipid accumulation by oleaginous yeasts grown on C-rich byproducts originated from food production and transformation has been observed [14–16]. More recently, the use of lignocellulosic biomass as raw carbon sources has been studied due to their high content on non-edible carbohydrates (i.e., cellulose and hemicellulose) and to their wide availability in nature [12, 17]. To allow yeast growth and lipid accumulation, lignocellulosic feedstocks need to be pre-treated by mechanical, physical and/or chemical methods to break the bonds among lignin, cellulose and hemicellulose. After this preliminary step, they can be easily hydrolyzed by enzyme cocktails to release free mono- and oligosaccharides. However, the above pre-treatment may sometimes generate variable amounts of lignin- and carbohydrate-derived byproducts (e.g., furfural and derivatives) that can inhibit yeast growth and metabolism [18–20]. Accordingly, the study of lipogenic ability of oleaginous yeasts on different pre-treated lignocellulosic feedstocks can be considered preparatory for developing sustainable biorefinery processes. Accordingly, some studies have been recently published [17, 20, 21]. However, some lignocellulosic feedstocks available in large quantities in the Mediterranean area, namely cardoon stalks (CS), stranded driftwood (SD) and residues from olive tree pruning (OTPR), have never been studied as carbon sources for lipid accumulation by oleaginous yeasts.

Cardoon (*Cynara cardunculus* L.) is a non-food oilseed crop exhibiting high growth rates (approx. 15 tons/ha × year) in arid soils with low fertilization requirements. About 12,000 tons/year of cardoon stalks are accumulated from Italian cultivations [22–24].

The accumulation of SD is an issue afflicting the Mediterranean coasts due to the intense rainfalls that transport downriver large volumes of wood trunks and soil sediments into the sea. During coastal storms these biomass feedstocks accumulate on the beaches, causing the formation of great (sometimes enormous) wood masses (about 200,000 tons/year on Italian shores) [25].

Olive tree (*Olea europaea* L.) is one of the most important fruit tree colonizing the Mediterranean area, which represents 98% of the world’s cultivated area of olive trees (approximately eight million hectares) [26]. In Italy alone, the amount of wood residues deriving from olive tree pruning is estimated over 1 million tons/year [27].

In total, CS, SD and OTPR constitute a huge amounts of residual biomass feedstocks that are still largely unexploited and give considerable issues due to the lack of cost-effective harvesting technology for their storage and disposal. Italian regulations classify most biomass feedstocks (e.g., SD) like to municipal solid wastes. So, local authorities have to manage their appropriate disposal, which is currently realized either through their costly storage into landfills (about 120€/ton) or through on-site burning. Accordingly, although the real possibility to realize an efficient and sustainable supply chain for feeding biorefineries is a still open question, the biotechnological use of these biomass feedstocks as cheap carbon sources is increasingly taken into consideration as an alternative environmental-friendly solution [25]. Therefore, some authors have suggested their use for producing biofuels and biochemicals [25, 28]. In the present study the ability of three basidiomycetous oleaginous yeasts, namely *Leucosporidium creatinivorum*, *Naganishia adeliensis* and *Solicoccozyma terricola*, to accumulate intracellular lipids in batch cultures on pre-treated cardoon stalks, stranded driftwood and residues from olive tree pruning has been studied.

## Methods

### Chemicals

Unless otherwise specified, all chemicals were from Sigma-Aldrich (Saint Louis, Missouri, USA) while all media were from Oxoid (Basingstoke, Hampshire, UK).

### Yeast strains

*Leucosporidium creatinivorum* DBVPG 4794, *Naganishia adeliensis* DBVPG 5195 and *Solicoccozyma terricola* DBVPG 5870 were used. They were previously selected on the basis of their superior lipogenic aptitude [13] and were preserved at − 80 °C in the Industrial Yeast Collection DBVPG of the Department of Agricultural, Food and Environmental Sciences, University of Perugia, Italy. Salient information on strains is reported on the DBVPG website (http://www.dbvpg.unipg.it). Working cultures were sub-cultured on YPD agar: 20 g/l glucose, 10 g/l yeast extract, 10 g/l peptone, 20 g/l agar, pH 6.0.

### Biomass feedstocks

CS were collected in 2015 after cardoon oilseeds harvesting and provided by Matrica S.p.A. (Porto Torres, Italy). SD was collected in 2015 in a 1000 m^2^ area close to the Italian Adriatic coast, by selecting wood pieces of different sizes to obtain a representative sample. OTPR were collected in 2015 in Italy after olive tree pruning. After collection, all above biomass feedstocks were stored at − 20 °C until use. The % of cellulose, hemicellulose and lignin of CS, SD and OTPR (before pre-treatments) are reported in Table 1.

**Table 1:** Composition of biomass feedstocks (before pre-treatment) used in this study; composition of water insoluble substrates (WIS) after steam-explosion; carbohydrates composition of WIS after steam-explosion and hydrolysis

	CS	SD	OTPR		
Composition of biomasses before pre-treatment (%)					
Cellulose	35.0 ± 1.5	31.4 ± 0.6	30.2 ± 0.2		
Hemicellulose	19.0 ± 1.1	14.9 ± 0.2	16.7 ± 0.1		
Xylan	14.0 ± 1.1	12.1 ± 0.1	12.5 ± 0.2		
Mannan	1.1 ± 0.1	1.2 ± 0.1	1.4 ± 0.2		
Galactan	1.6 ± 1.1	0.6 ± 0.2	0.9 ± 0.1		
Arabinian	2.2 ± 0.2	1.0 ± 0.2	1.8 ± 0.1		
Lignin	29.4 ± 1.7	27.8 ± 1.7	21.0 ± 1.4		

	CS	SD1	SD2	OTPR1	OTPR2
Composition of water insoluble substrate (WIS) after steam-explosion (%)					
Cellulose	54.0 ± 1.6	44.3 ± 0.3	46.8 ± 0.1	49.0 ± 0.8	44.7 ± 0.2
Hemicellulose	6.0 ± 0.2	5.8 ± 0.1	0.8 ± 0.1	4.9 ± 0.1	3.1 ± 0.1
Lignin	32.8 ± 0.7	44.0 ± 1.2	47.1 ± 0.1	38.9 ± 0.9	44.3 ± 0.6
Carbohydrates composition of hydrolyzed WIS obtained after enzymatic hydrolysis (g/l)					
Glucose	38.7 ± 0.9	40.6 ± 0.2	44.8 ± 0.2	40.5 ± 0.1	37.3 ± 0.2
Xylose	4.7 ± 0.3	5.1 ± 0.1	0.5 ± 0	3.6 ± 0.1	3.1 ± 0.1
Cellobiose	1.1 ± 0.1	1.3 ± 0.1	1.7 ± 0.1	1.7 ± 0.1	1.8 ± 0.1
WIS enzymatic hydrolysis yields (%)					
Glucose	78.7 ± 1.5	55.0 ± 0.2	63.2 ± 0.2	44.6 ± 0.3	55.2 ± 0.2

Cellulose enzymatic hydrolysis yields calculated after steam-explosion and hydrolysis. *CS* cardoon stalks, *SD* stranded driftwood, *OTPR* olive tree pruning residues

### Pre-treatment of biomass: steam-explosion

All biomass feedstocks were preliminarily dried at 40 °C for 1 week and then subjected to size reduction (min. 2 mm, max. 3 cm) by a cutting mill. Steam-explosion of biomass was performed to deconstruct the lignocellulosic portion making it accessible to hydrolytic enzymes.

Steam-explosion of CS was conducted into a 10 l batch reactor (Stake Tech-Norval, Ontario, Canada) as reported by Liuzzi et al. [29]. Briefly, biomass was firstly soaked in a 0.6% H_2_SO_4_ solution for 10 min and, therefore, the solid portion was separated from the solution. The acid concentration was settled on the basis of the final variation of dry weight (DW, from 81 to 35–40%) of CS after acid soaking. The SE process was preliminarily optimized by reaching the final conditions: 195 °C, 7.5 min [29].

Steam-explosion of both SD and OTPR was conducted into a 11 l batch reactor (Biochemtex, Tortona, Italy) with no chemical hydrolysis at two different optimized conditions [25]. Briefly, steam-explosion of SD was performed either at 190 °C for 25 min (the resulting fraction was labeled as SD1) and at 210 °C for 25 min (SD2), while steam-explosion of OTPR was performed either at 190 °C for 40 min (the resulting fraction was labeled as OTPR1) and at 210 °C for 25 min (OTPR2).

Pre-treatment of lignocellulosic feedstocks via steam-explosion released two different fractions: (i) a water insoluble substrate (WIS) containing a mixture of cellulose and lignin; and (ii) a pre-treatment liquor (PL) containing hemicellulose, C5 carbohydrates and some inhibitors, which need to be detoxified for allowing microbial growth and metabolism. The WIS was separated from PL by a stainless steel filter (cutoff 1 mm), washed with water at 50 °C for 30 min using a solid/liquid (S/L) ratio of 10% (w/w) [30] and then analyzed for their content of cellulose, hemicellulose and lignin following the National Renewable Energy Laboratory (NREL) analytical procedures [31]. Briefly, acid hydrolysis with H_2_SO_4_ of each sample was performed in triplicate to obtain C5 and C6 monomers from cellulose and hemicellulose. The concentration of both C5 and C6 monomers was detected by Dionex Ultimate 3000 HPLC (Thermo Scientific, Sunnyvale, CA, USA) equipped with a Biorad Aminex HPX-87H column (Biorad, California, USA) thermo-regulated at 50 °C and a RI detector (RefractoMax520, Thermo Scientific, Waltham, MA USA), mobile phase = 0.01 N H_2_SO_4_, flow 0.6 ml/min. The concentration of polymeric sugars was calculated using an anhydrous correction of 0.88 and 0.90 for C5 and C6 carbohydrates, respectively. The remaining acid-insoluble residue was used to calculating the acid-insoluble lignin after removing the ash content. The % of cellulose, hemicellulose and lignin of WIS of CS, SD and OTPR after steam-explosion are reported in Table 1.

### Pre-treatment of biomass: enzymatic hydrolysis of WIS

The WIS was selected for the subsequent phase of enzymatic hydrolysis to release mono- and oligosaccharides from cellulose, due to their higher contents of carbohydrates, as suggested by current literature [25, 30, 32–34].

WIS of CS (S/L ratio of 8% w/w) was hydrolyzed for 72 h at pH 5 and 50 °C in a 1.5 l Biostat B stirred bioreactor (B. Braun Biotech International, Walpole, MA, USA). An enzyme cocktail solution (CTEC2, Novozyme, Denmark) with an activity of 150 FPU/ml and 5444 CBU/ml and a density of 1.3 g/ml was used with a dosage of 190 mg/g of insoluble glucans.

On the other hand, WIS of SD1 and SD2, and OTPR1 and OTPR2 (S/L ratio of 15% w/w) were hydrolyzed for 95 h at pH 5 and 50 °C in a 5 l Biostat^®^ A-Plus-Sartorius stirred bioreactor (Sartorius, Goettingen, Germany). An enzyme cocktail solution (NS-22192, Novozyme, Denmark) with an activity of 120 FPU/ml and 4500 CBU/ml and a density of 1.2 g/ml was used with a dosage of 150 mg/g of insoluble glucans. The enzymatic activity was determined by NREL standard procedure [35] and as previously reported [36, 37]. Both bioreactors are equipped with an automatic monitoring and controlling system for rotation speed, pH, aeration, temperature and antifoam.

After hydrolysis, the hydrolyzates were heat-treated to quench the residual enzymes activity. The solid–liquid separation following enzymatic hydrolysis was thus performed: insoluble residual lignin fraction was separated from the carbohydrate-rich hydrolyzed liquid fraction by filtration (cutoff 0.45 µm) under pressure (73 g/m^2^).

The concentration of glucose, xylose, and cellobiose on hydrolyzed CS, SD1, SD2, OTPR1 and OTPR2 was determined by HPLC as shown in the previous paragraph. Results are reported in Table 1. The yield of the enzymatic hydrolysis of WIS (C_Hy %_) was calculated as shown in Eq. 1 [38] considering the transformation of cellulose into glucose.

1 $${\text{C}}_{{{\text{Hy}}\%}} = \left[ {{{\left( {r_{{{\text{Gc}}}} f_{{\text{G}}} } \right)} \mathord{\left/ {\vphantom {{\left( {r_{{{\text{Gc}}}}\;f_{{\text{G}}} } \right)} {\left( {{\text{WIS}}_{{\text{l}}}\;{\text{C}}_{\% } } \right)}}} \right. \kern-\nulldelimiterspace} {\left( {{\text{WIS}}_{{\text{l}}} {\text{C}}_{\% } } \right)}}} \right]\;{10}^{4}$$

where *r*_Gc_ = the anhydrous glucose correction (0.90); *f*_G_ = grams of glucose mass fraction (found into the slurry at the end of hydrolysis); WIS_l_ = grams of water insoluble substrate loaded into the bioreactor; C_%_ = the percentage of cellulose found in the WIS (Table 1).

To calculate the C/N ratio, the total nitrogen content of hydrolyzed CS, SD1, SD2, OTPR1 and OTPR2 was determined by semi-micro Kjeldahl method as described in AOAC Official Methods^SM^ [39].

After hydrolysis all CS, SD and OTPR samples were stored at − 20 °C until use.

### Shaken flask batch cultures (lipid accumulation tests)

Batch cultures were carried out at 20 or 25 °C to check the influence of temperature on yeast lipogenic performances. A loopful of 48 h cells of each yeast strains grown on YPD agar was inoculated in 50 ml orbital shaken flasks (160 rpm) containing 10 ml of pre-culture medium (50% of YPD broth and 50% of steam-exploded and hydrolyzed CS, SD1, SD2, OTPR1 or OTPR2. The pH of pre-culture media was adjusted to 5.5 with NaOH 1 M and yeast extract was added to obtain a C/N ratio of about 40. After incubation at 20 or 25 °C for 24 h, 1 ml of each pre-culture (A_600_ adjusted to 0.1) was inoculated in 100 ml orbital shaken flasks (160 rpm) containing 20 ml of steam-exploded and hydrolyzed CS, SD1, SD2, OTPR1 or OTPR2. As above, pH was adjusted to 5.5 with NaOH 1 M and yeast extract was added to obtain a C/N ratio of about 40. Samples were incubated at 20 or 25 °C until the complete depletion of carbohydrates.

During batch cultivation, yeasts growth was monitored spectrophotometrically (Beckman DU^®^ 640, Brea, CA, USA) by measuring A_600_, while carbohydrate depletion was checked by enzymatic commercial kits: K-GLUC 07/11 (glucose), K-XYLOSE 08/14 (xylose), K-ARGA 02/15 (galactose) (Megazyme, Chicago, IL, USA) by following the protocols indicated by the supplier. Cellobiose depletion was monitored as reported by Filippucci et al. [13]. Briefly, the commercial β-glucosidase supplied with E-BGOSAG kit (Megazyme) was diluted 1:10 in 50 mM sodium maleate buffer (pH 6.5) in the presence of 0.5 mg/ml of Bovine Serum Albumin. After incubation (40 °C for 15 min) the quantification of glucose released by cellobiose hydrolysis was carried out using K-GLUC 07/11.

The amount (g/l) of yeast biomass produced after batch incubations was determined gravimetrically as cell DW [13].

### Extraction of intracellular lipids

The extraction of intracellular lipids was performed using the protocol reported by Filippucci et al. [13]. Briefly, 10 ml of each culture was centrifuged (5000×*g* for 10 min) and repeatedly washed with distilled water. The cells were thus treated with 10 ml of 4 M HCl, incubated at 60 °C for 2 h in a water bath to obtain acid-hydrolyzed cells, mixed with 15 ml of a chloroform/methanol 2:1 (v/v) mixture and incubated at room temperature for 2 h in an orbital shaker at 160 rpm. After incubation, the samples were centrifuged (4000 rpm for 10 min) to obtain the separation of the different phases. The organic phase containing the lipids was separated and put inside glass vials which were fluxed to dryness in the dark by a gas nitrogen flow. Glasses were then instantly sealed with a rubber septum, weighed to determine the total amount of lipids and stored at − 20 °C until GC–MS analysis.

The weight of lipids extracted from yeast cells, the amount of yeast biomass produced after batch cultures, the content of glucose, xylose and cellobiose of the hydrolyzed biomass, and the duration of incubation required for obtaining the complete depletion of carbohydrates were used to calculate the following parameters: (i) the total lipid production (P_L_, g/l); (ii) % of total intracellular lipid on cell biomass (P_L_/DW); (iii) the lipid yield (P_L_/C = ratio between the total lipid production and the amount of carbohydrates used by yeasts for growth and metabolism); and (iv) the daily productivity [P_L_/d, g/(l × day)].

### Determination of fatty acid profiles by GC–MS

The determination of fatty acid (FA) profiles was performed as reported by Rossi et al. [40] with a few modifications. Briefly, dried lipids stored into glass vials were dissolved in 4 ml of a 2:1:1 mixture of chloroform, boron trifluoride alcoholic solution (10% methanol) and 2,2-dimethoxypropane (acting as water scavenger) and transferred into a Schlenk tube. Glyceryl triundecanoate was added to the reaction mixture to generate the internal standard for GC–MS analysis. Trans-esterification was carried out at 55 °C for 1 h. Analysis was performed using a quadrupole GC–MS system (6890 N GC + 5795B MS detector) equipped with an EI ionization detector (70 eV ionization energy) (Agilent, Santa Clara, CA, USA). An OMEGAWAX GC capillary column (length 30 m, internal diameter 0.25 mm, film thickness of 0.25 µm (SUPELCO—Bellefonte, PA, USA) was used for the separation of the different FA. The injection temperature was 250 °C and the oven temperature was programmed as follows: (i) an isotherm at 50 °C for 2 min; (ii) a gradient (4 °C/min) from 50 to 220 °C; and (iii) a final isotherm of 18 min at 220 °C. High-purity hydrogen was used as mobile phase and a constant flow of 1.2 ml/min was maintained during the analysis. FA profiles were identified by comparing their retention times with those of commercial standards of fatty acyl methyl esters (FAMEMix 37, Sigma-Aldrich). Peak areas in the total ion chromatograms were used to determine their relative amounts.

The Watson’s Eq. (2) was used to calculate the unsaturation index (UI) of lipids extracted from yeasts [41]:

2 $${\text{UI}} = \, \left[ {\% {\text{ monoenes }} + { 2}\left( {\% {\text{ dienes}}} \right) \, + { 3}\left( {\% {\text{ trienes}}} \right)} \right]/ 100$$

### Statistical analysis

Batch fermentations were carried out in triplicate, and, wherever necessary, statistical testing was performed using ANOVA. Generalized least squares were used and, whenever necessary, a different standard deviation was allowed for each predictor level, to account for heteroscedasticity [42]. Means were compared using Fisher LSD (Tukey HSD) [43].

Principal Component Analyses (PCA) were carried out on the FA profiles using the R environment for statistical computing [44]. Data were not standardized prior to analysis and results relating to the main FA were displayed on correlation biplots [45]. PCA were still performed on the standardized percentages of saturated FA (SFA), unsaturated FA (UFA) and unsaturation index (UI). Results were also displayed on correlation biplots.

## Results and discussion

### Biomass composition before and after the pre-treatment

The three feedstocks investigated exhibited some differences in the chemical composition before the pre-treatment, mainly in the terms of cellulose and lignin. About the hemicellulose, the main component was xylose (> 73% for all the feedstocks) (Table 1). After pre-treatment the WIS showed different compositions due to the different process conditions (i.e., temperature, process duration, and acid catalyst) which gave a differential degradation of both cellulose and hemicellulose. The highest degradation was found in SD2. CS retained the highest content of cellulose (54.0%) while, on the contrary, a more similar composition in terms of cellulose and lignin was found in SD and OTPR (Table 1). The highest enzymatic hydrolysis yields (in terms of released glucose) were found in CS and SD2 (Table 1).

### Lipid accumulation by oleaginous yeasts on CS, SD1, SD2, OTPR1 and OTPR2

P_L_, DW, P_L_/DW, P_L_/C and P_L_/d of *L. creatinivorum* DBVPG 4794, *N. adeliensis* DBVPG 5195 and *S. terricola* DBVPG 5870 grown on of steam-exploded and hydrolyzed CS, SD1 and SD2, and OTPR1 and OTPR2 at 20 and 25 °C are reported in Table 2.

**Table 2:** Lipogenic aptitude of *Leucosporidium creatinivorum* DBVPG 4794, *Naganishia adeliensis* DBVPG 5195 and *Solicoccozyma terricola* DBVPG 5870 grown on steam-exploded and hydrolyzed CS (cardoon stalks), SD1, SD2 (stranded driftwood), OTPR1 and OTPR2 (olive tree pruning residues) at 20 and 25 °C

	T (°C)	P_L_ (g/l)	DW (g/l)	P_L_/DW (%)	P_L_/C (%)	P_L_/d [g/(l × day)]
CS						
*Naganishia adeliensis* DBVPG 5195	20	6.87 ± 0.2^fgh^	13.90 ± 0.4^cd^	49.33 ± 0.5^de^	15.06 ± 0.5^efgh^	1.00 ± 0^f^
	25	6.59 ± 0.2^efg^	14.94 ± 0.3^de^	44.10 ± 0.9^c^	14.45 ± 0.5^defg^	0.47 ± 0^bcd^
*Solicoccozyma terricola* DBVPG 5870	20	13.20 ± 0.2^l^	23.75 ± 0.2^i^	55.60 ± 0.7^g^	28.95 ± 0.4^m^	1.70 ± 0^j^
	25	10.22 ± 0.5^k^	19.10 ± 1.0^h^	53.60 ± 4.4^cdefg^	22.41 ± 1.1^l^	1.36 ± 0.6^i^
SD1						
*Leucosporidium creatinivorum* DBVPG 4794	20	9.20 ± 0.4^jk^	16.70 ± 0.2^fg^	55.10 ± 2.7^defg^	19.57 ± 0.8^jkl^	1.03 ± 0.1^f^
*Naganishia adeliensis* DBVPG 5195	20	7.07 ± 0.5^efghi^	12.43 ± 0.1^abc^	56.63 ± 4.0^defg^	15.03 ± 1.1^bcdefgh^	1.17 ± 0.1^gh^
	25	8.19 ± 0.7^ij^	14.45 ± 0.5^de^	56.70 ± 2.8^fg^	17.43 ± 1.5^hij^	0.51 ± 0^d^
SD2						
*Leucosporidium creatinivorum* DBVPG 4794	20	9.83 ± 0.5^k^	17.92 ± 0.1^gh^	54.88 ± 3.0^defg^	20.95 ± 1.2^kl^	1.23 ± 0.1^h^
*Naganishia adeliensis* DBVPG 5195	20	6.65 ± 0.1^fg^	12.50 ± 0.5^abc^	53.27 ± 1.8^efg^	14.17 ± 0.1^def^	1.10 ± 0^fg^
	25	8.50 ± 1.1^hijk^	14.35 ± 0.6^de^	59.13 ± 4.9 ^fg^	18.11 ± 2.3^ghijk^	0.53 ± 0.1^d^
*Solicoccozyma terricola* DBVPG 5870	20	9.87 ± 0.1^k^	18.47 ± 0.8^h^	53.50 ± 2.0^defg^	21.02 ± 0.1^kl^	1.63 ± 0.1^j^
	25	7.78 ± 0.6^ghij^	14.37 ± 0.6^de^	54.10 ± 3.5^defg^	16.57 ± 1.2^fghij^	0.49 ± 0^cd^
OTPR1						
*Leucosporidium creatinivorum* DBVPG 4794	20	6.22 ± 0.4^cdef^	12.10 ± 1.6^ab^	51.60 ± 3.5^defg^	13.60 ± 0.8^bcde^	0.35 ± 0.1^ab^
	25	5.87 ± 0^c^	14.60 ± 0.3^de^	40.19 ± 0.9^b^	12.83 ± 0^b^	0.27 ± 0^a^
*Naganishia adeliensis* DBVPG 5195	20	6.27 ± 0.2^de^	12.70 ± 0.3^bc^	49.39 ± 0.2^d^	13.72 ± 0.4^cd^	0.57 ± 0^d^
	25	5.90 ± 0.2^bcd^	18.30 ± 0.3^h^	32.24 ± 0.3^a^	12.91 ± 0.5^bc^	0.74 ± 0^e^
*Solicoccozyma terricola* DBVPG 5870	20	8.15 ± 0^i^	15.50 ± 0.1^ef^	52.55 ± 0.4^f^	17.82 ± 0^i^	0.74 ± 0^e^
OTPR2						
*Naganishia adeliensis* DBVPG 5195	20	4.90 ± 0.1^a^	11.04 ± 0.1^a^	44.38 ± 0.6^c^	11.60 ± 0.3^a^	0.38 ± 0^abc^
	25	5.58 ± 0.1^b^	12.50 ± 0^abc^	44.63 ± 1.1^c^	13.22 ± 0.3^bc^	0.51 ± 0^d^

*P*_*L*_ total lipid production, *DW* cell dry weight, *P*_*L*_*/DW* % of total intracellular lipid on cell biomass, *P*_*L*_*/C* lipid yield, *P*_*L*_*/d* daily productivity

Different superscripted letters indicate significant (p < 0.05) different values, as assessed by Tukey HSD [43]

None of the three tested biomass feedstocks supported both growth and lipid accumulation by all three yeasts (Table 2). Some significant (p < 0.05) differences were found in the results (i.e., P_L_, DW, P_L_/DW, P_L_/C and P_L_/d): overall these differences were related to the different strains, different biomass feedstocks, different incubation temperatures or even a combination of these (Table 2). Overall, *N. adeliensis* was the most versatile strain: it was able to growth at both 20 and 25 °C on all substrates although its lipogenic aptitude was generally lower to those exhibited by the other yeasts (Table 2), as confirmed by Li et al. [46], who reported the capacity of this species to produce intracellular lipids up to 33.1%. On the other hand, *S. terricola* grown on CS was the most productive strain: it displayed significantly (p < 0.05) higher results at both 20 °C and 25 °C, both in terms of P_L_ (13.20 and 10.22 g/l, respectively) and P_L_/C (28.95 and 22.41%) (Table 2). Interestingly the P_L_/C exhibited by *S. terricola* grown on CS at 20 °C was close to the maximum P_L_/C theoretical value (31.6%) [47].

OTPR1 and OTPR2 supported lower lipogenic aptitudes by all three strains; in particular, P_L_/d was always below to 0.75 g/(l × day) (Table 2). This lower performance could be due to the presence of some phenolic compounds in olive wood, which might act as inhibitors of yeast growth and metabolism. This hypothesis is consistent with a previous study that reports the presence of a few antimicrobial compounds, namely hydroxytyrosol, tyrosol, cycloolivil, 7-deoxyloganic acid and oleuropein in olive tree wood extracts [48].

*Solicoccozyma terricola* always exhibited a P_L_/DW over 50% at both 20 and 25 °C (Table 2), thus confirming a previous study [49] reporting that this yeast species is able to accumulate high percentages of intracellular lipids. *L. creatinivorum* and *N. adeliensis* exhibited some significant (p < 0.05) values of P_L_/DW in dependence of the different substrates and incubation temperatures, or a combination of both (Table 2). These trends confirmed that the increase of P_L_/DW was not always correlated to a proportional increase of P_L_, as previously suggested [13, 50].

The relationships between the intracellular lipid contents (P_L_/DW) and the incubation temperature were found only in *L. creatinivorum* and *N. adeliensis* grown on OTPR1, and CS and OTPR1, respectively, they exhibited a significant (p < 0.05) increase of P_L_/DW as the consequence of the decrease from 25 to 20 °C (Table 2). The lipogenic aptitude of *L. creatinivorum* was described in some recent studies: a percentage of lipid on cell biomass from 60 to 70% was reported [13, 51, 52].

### Fatty acid profiles of lipids produced by oleaginous yeasts

The FA profiles of *L. creatinivorum*, *N. adeliensis* and *S. terricola* grown on of steam-exploded and hydrolyzed CS, SD1 and SD2, and OTPR1 and OTPR2 at 20 and 25 °C are reported in Table 3. Overall, the main FA were palmitic (hexadecanoic acid = C16:0), stearic (octadecanoic acid = C18:0), oleic [(9E9Z)-octadec-9-enoic acid = Δ9C18:1], linoleic [(9Z,12Z)-9,12-octadecadienoic acid = ∆9,12C18:2], α-linolenic [(9Z,12Z,15Z)-9,12,15-octadecatrienoic acid = ∆9,12,15C18:3] and γ-linolenic [(6Z,9Z,12Z)-6,9,12-octadecatrienoic acid = ∆6,9,12C18:3] acids. Other minor FA, namely caproic (hexanoic acid = C6:0), caprylic (octanoic acid = C8:0), capric (decanoic acid = C10:0), lauric (dodecanoic acid = C12:0), myristic (tetradecanoic acid = C14:0), palmitoleic [(9Z)-hexadec-9-enoic acid = ∆9C16:1), arachic (eicosanoic acid = C20:0), gondoic [(11Z)-11-eicosenoic acid = ∆11C20:1], behenic (docosanoic acid = C22:0), erucic [(13Z)-docos-13-enoic acid = ∆13C22:1] and lignoceric (tetracosanoic acid = C24:0) acids were also found (Table 3).

**Table 3:** Fatty acid (FA) profiles of lipids produced by *Leucosporidium creatinivorum* DBVPG 4794, *Naganishia adeliensis* DBVPG 5195 and *Solicoccozyma terricola* DBVPG 5870 grown on steam-exploded and hydrolyzed CS (cardoon stalks), SD1, SD2 (stranded driftwood), OTPR1 and OTPR2 (olive tree pruning residues) at 20 and 25 °C

	T (°C)	C6:0 (%)	C8:0 (%)	C10:0 (%)	C12:0 (%)	C14:0 (%)	C16:0 (%)	C16:1 (%)	C18:0 (%)	C18:1 (%)
CS										
*Naganishia adeliensis* DBVPG 5195	20	0	0	0.01 ± 0	0.02 ± 0	0.54 ± 0.1	19.94 ± 2.2^fghij^	0.05 ± 0	9.95 ± 0.8^gh^	63.78 ± 2.0^fg^
	25	0	0	0.01 ± 0	0.01 ± 0	0.69 ± 0.1	16.15 ± 1.5^cdefg^	0	13.48 ± 1.1^i^	63.17 ± 1.1^f^
*Solicoccozyma terricola* DBVPG 5870	20	0.01 ± 0	0.01 ± 0	0.02 ± 0	0.02 ± 0	0.41 ± 0.1	31.88 ± 0.2^m^	0.54 ± 0.1	12.59 ± 0.5^i^	48.29 ± 0.6^c^
	25	0	0.01 ± 0	0.03 ± 0	0.02 ± 0	0.41 ± 0.1	38.22 ± 6.4^n^	0.42 ± 0.1	25.25 ± 7.0^hijk^	29.23 ± 2.2^a^
SD1										
*Leucosporidium creatinivorum* DBVPG 4794	20	0.02 ± 0	0	0	0	0	17.78 ± 0.4^gh^	0.7 ± 0.2	3.71 ± 0.6^ab^	65.70 ± 1.9^fg^
*Naganishia adeliensis* DBVPG 5195	20	0.1 ± 0	0	0	0	0	26.18 ± 0.9^kl^	0.07 ± 0	12.65 ± 1.3^i^	51.83 ± 2.2^cd^
	25	0.05 ± 0	0	0	0	0	24.07 ± 4.4^hijkl^	0.07 ± 0	7.52 ± 1.4^defg^	59.42 ± 5.9^defg^
SD2										
*Leucosporidium creatinivorum* DBVPG 4794	20	0.02 ± 0	0	0	0	0.99 ± 0	18.04 ± 2.1^defghi^	0.81 ± 0.1	3.09 ± 0.3^a^	68.87 ± 2.1^gh^
*Naganishia adeliensis* DBVPG 5195	20	0.13 ± 0.1	0	0	0.03 ± 0	0.18 ± 0.1	11.29 ± 1.9^bc^	0	7.98 ± 1.0^efg^	74.13 ± 2.5^hi^
	25	0.03 ± 0	0	0	0	0.21 ± 0	6.62 ± 0.6^a^	0.17 ± 0	8.71 ± 0.8^efg^	77.61 ± 1.9^i^
*Solicoccozyma terricola* DBVPG 5870	20	0	0	0	0	0.2 ± 0.1	23.63 ± 2.4^ijkl^	0.57 ± 01	11.30 ± 1.5^jhi^	58.60 ± 0.7^e^
	25	0	0	0.03 ± 0	0	1.09 ± 0.1	29.47 ± 3.9^jklm^	0.15 ± 0.1	24.03 ± 1.0 ^k^	41.12 ± 3.2^b^
OTPR1										
*Leucosporidium creatinivorum* DBVPG 4794	20	0	0	0	0.24 ± 0.2	1.57 ± 0.3	16.52 ± 0.4^f^	0.58 ± 0.3	4.81 ± 0.6^bc^	68.11 ± 1.5^gh^
	25	0	0	0	0.73 ± 0.3	3.16 ± 0.9	15.74 ± 0^f^	0.61 ± 0.2	8.92 ± 0.6^fg^	62.38 ± 2.3^efg^
*Naganishia adeliensis* DBVPG 5195	20	0	0	0	0.44 ± 0	1.69 ± 0.5	15.85 ± 0.4^ef^	0.56 ± 0.2	8.70 ± 0.4^fg^	65.23 ± 1.7^fg^
	25	0	0	0	0.53 ± 0.1	1.98 ± 0.9	13.76 ± 0.7^bcd^	0.44 ± 0.1	7.75 ± 0.5^ef^	69.29 ± 3.6^fghi^
*Solicoccozyma terricola* DBVPG 5870	20	0	0	0	0.29 ± 0	1.03 ± 0.1	24.13 ± 0.8^jk^	0.41 ± 0	18.70 ± 0.6^j^	51.80 ± 0.5^d^
OTPR2										
*Naganishia adeliensis* DBVPG 5195	20	0	0	0	0.67 ± 0.1	2.71 ± 0.9	14.66 ± 0.4^cde^	0.56 ± 0.1	7.47 ± 0.2^e^	66.92 ± 1.4^fg^
	25	0	0	0	0.45 ± 0	2.11 ± 1	11.60 ± 0.8^b^	0.34 ± 0.1	5.37 ± 0.4 ^cd^	76.53 ± 2.5^i^

	T (°C)	C18:2 (%)	C18:3 n − 6 (%)	C18:3 n − 3 (%)	C20:0 (%)	C20:1 (%)	C22:0 (%)	C22:1 (%)	C24:0 (%)
CS									
*Naganishia adeliensis* DBVPG 5195	20	5.08 ± 0.6^cd^	0.02 ± 0	0.13 ± 0.1	0.12 ± 0.1	0.03 ± 0	0.18 ± 0.1	0	0.14 ± 0
	25	5.39 ± 0.3^d^	0.01 ± 0	0.26 ± 0	0.33 ± 0.2	0.06 ± 0	0.23 ± 0.1	0	0.21 ± 0.2
*Solicoccozyma terricola* DBVPG 5870	20	4.67 ± 0.5^cd^	0.03 ± 0	0.11 ± 0	0.45 ± 0	0.07 ± 0	0.36 ± 0	0	0.54 ± 0.3
	25	5.22 ± 1.4^abcde^	0.01 ± 0	0.15 ± 0	0.52 ± 0.1	0.02 ± 0	0.27 ± 0.1	0	0.21 ± 0.2
SD1									
*Leucosporidium creatinivorum* DBVPG 4794	20	8.30 ± 1.1^efg^	0.06 ± 0	2.42 ± 0.1	0.48 ± 0.1	0.19 ± 0.1	0.44 ± 0.1	0.21 ± 0.1	0
*Naganishia adeliensis* DBVPG 5195	20	9.17 ± 0.1^g^	0	0	0	0	0	0	0
	25	8.62 ± 0.1^f^	0	0	0.17 ± 0	0.09 ± 0	0	0	0
SD2									
*Leucosporidium creatinivorum* DBVPG 4794	20	8.19 ± 0.4^ef^	0	0	0	0	0	0	0
*Naganishia adeliensis* DBVPG 5195	20	4.84 ± 2.0^abcdefg^	0	0	0.75 ± 1.2	0.56 ± 0.9	0	0	0
	25	6.54 ± 3.3^abcdefg^	0.04 ± 0	0.07 ± 0	0	0	0	0	0
*Solicoccozyma terricola* DBVPG 5870	20	3.38 ± 0.6^abc^	0.18 ± 0	0.07 ± 0	0.83 ± 0.1	0.08 ± 0	0.46 ± 0.1	0.21 ± 0.1	0.45 ± 0.2
	25	3.44 ± 0.1^b^	0.07 ± 0	0.10 ± 0	0.37 ± 0	0.03 ± 0	0	0	0
OTPR1									
*Leucosporidium creatinivorum* DBVPG 4794	20	3.80 ± 0.2^bc^	2.64 ± 1.2	0.32 ± 0.1	0.46 ± 0.3	0	0	0	0
	25	5.66 ± 0.1^d^	1.06 ± 0.2	0.43 ± 0.1	1.29 ± 0.4	0	0	0	0
*Naganishia adeliensis* DBVPG 5195	20	5.42 ± 0.2^d^	0.93 ± 0	0.37 ± 0	0.84 ± 0	0	0	0	0
	25	3.25 ± 0.2^b^	0.58 ± 0.1	0.45 ± 0.1	0.97 ± 0.1	0	0	0	0
*Solicoccozyma terricola* DBVPG 5870	20	2.41 ± 0.2^a^	0.56 ± 0.2	0.34 ± 0	0.33 ± 0	0	0	0	0
OTPR2									
*Naganishia adeliensis* DBVPG 5195	20	4.43 ± 0.2^c^	0.87 ± 0.2	0.43 ± 0	1.27 ± 0.2	0	0	0	0
	25	2.21 ± 0.2^a^	0.30 ± 0	0.34 ± 0	0.75 ± 0.1	0	0	0	0

*C6:0* caproic acid (hexanoic acid), *C8:0* caprylic acid (octanoic acid), *C10:0* capric acid (decanoic acid), *C12:0* lauric acid (dodecanoic acid), *C14:0* myristic acid (tetradecanoic acid), *C16:0* palmitic acid (hexadecanoic acid), *∆9C16:1* palmitoleic acid [(9Z)-hexadec-9-enoic acid), *C18:0* stearic acid (octadecanoic acid), *Δ9C18:1* oleic acid [(9E9Z)-octadec-9-enoic acid], *∆9,12C18:2* linoleic acid [(9Z,12Z)-9,12-octadecadienoic acid], *∆9,12,15C18:3* α-linolenic acid [(9Z,12Z,15Z)-9,12,15-octadecatrienoic acid], *∆6,9,12C18:3* γ-linolenic acid [(6Z,9Z,12Z)-6,9,12-octadecatrienoic acid], *C20:0* arachic acid (eicosanoic acid), *∆11C20:1* gondoic acid [(11Z)-11-eicosenoic acid], *C22:0* behenic acid (docosanoic acid), *∆13C22:1* erucic acid [(13Z)-docos-13-enoic acid], *C24:0* lignoceric acid (tetracosanoic acid)

For the fatty acids C16:0, C18:0, C18:1 and C18:2 different superscripted letters indicate significant (p < 0.05) different values, as assessed by Tukey HSD [43]

Also in this case, some significant (p < 0.05) differences were found among the percentages of the main FA (i.e., palmitic, stearic, oleic and linoleic acids) in relation to the different strains, different biomass feedstocks, different incubation temperatures or even a combination of these. Overall, oleic acid was the dominant FA found in lipids produced by the three yeasts (from 29.23 to 77.61%). This is in agreement with the current literature reporting oleic acid as the principal FA (from about 40 to 80% of total FA) produced by oleaginous yeasts [8, 12, 13, 50]. The sole exception to this general trend was the FA profile of *S. terricola* grown on CS at 25 °C, which exhibited a significantly (p < 0.05) higher % of palmitic acid (38.22%) (Table 3). However, we cannot consider this result surprising, because other FA other than oleic acid was sometimes found to be dominant: previous studies reported that batch cultures of *Lipomyces starkeyi*, *Yarrowia lipolytica, Vanrija* (former *Cryptococcus*) *humicola* and *Rhodotorula* (former *Rhodosporidium*) *toruloides* grown on different carbon sources exhibited linoleic or palmitic acids as main FA [16, 53].

**Fig. 1:**
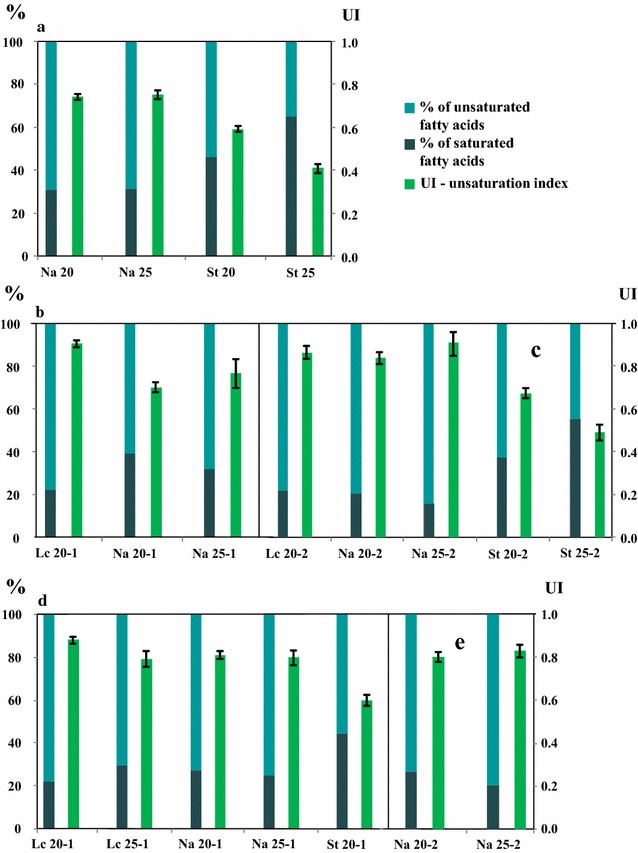
% of unsaturated and saturated fatty acids (UFA and SFA), and unsaturation index (UI) of lipids produced by *Leucosporidium creatinivorum* DBVPG 4794, *Naganishia adeliensis* DBVPG 5195 and *Solicoccozyma terricola* DBVPG 5870 grown on steam-exploded and hydrolyzed CS (cardoon stalks), SD1, SD2 (stranded driftwood), OTPR1 and OTPR2 (olive tree pruning residues) at 20 and 25 °C. **a** Na 20 and Na 25, and St 20 and St 25 = *N. adeliensis* and *S. terricola*, respectively, grown on CS at 20 and 25 °C; **b**, **c** Lc 20-1 and Lc 20-2 = *L. creatinivorum* grown on SD1 and SD2, respectively, incubated at 20 °C; Na 20-1, Na 20-2, Na 25-1 and Na 25-2 = *N. adeliensis* grown on SD1 and SD2, respectively, incubated at 20 and 25 °C; St 20-2 and St 25-2 = *S. terricola* grown on SD2, incubated at 20 and 25 °C; **d**, **e** Lc 20-1 and Lc 25-1 = *L. creatinivorum* grown on OTPR1, incubated at 20 and 25 °C; Na 20-1, Na 20-2, Na 25-1 and Na 25-2 = *N. adeliensis* grown on OTPR1 and OTPR2, respectively, incubated at 20 and 25 °C; St 20-1 = *S. terricola* grown on OTPR1, incubated at 20 °C

Overall, *L. creatinivorum* exhibited always a % of UFA up to 70%, while the highest values of  % of UFA and UI were found in *N. adeliensis* grown on SD2 at 25 °C (close 85% and 0.90 respectively) (Fig. 1).

*Solicoccozyma terricola* grown on CS (Fig. 1a) and SD2 (Fig. 1c) and *L. creatinivorum* on OTPR1 (Fig. 1d) exhibited a significant (*p *< 0.01) increase of both UFA/SFA ratio and UI when the incubation temperature decreased from 25 to 20 °C, in agreement with current literature reporting an inverse relationship between UFA/SFA ratio and UI, and incubation temperature [13, 40, 41]. However, even though the change of lipid metabolism is a well-established physiological adaptation strategy adopted by both psychrophilic and psychrotolerant yeasts when the growth temperature decreases [5, 13, 40, 41], this trend was not observed in all strains (Fig. 1).

**Fig. 2:**
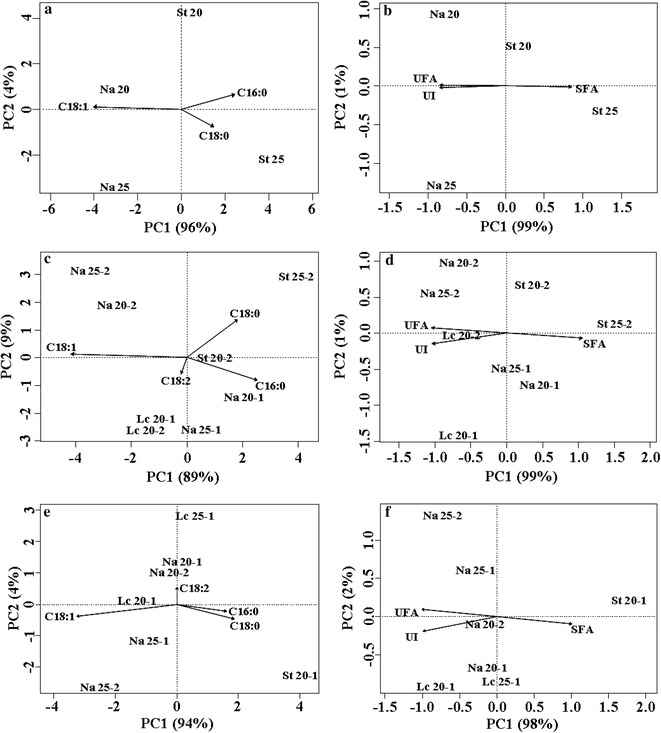
PCA of lipids produced by *Leucosporidium creatinivorum* DBVPG 4794, *Naganishia adeliensis* DBVPG 5195 and *Solicoccozyma terricola* DBVPG 5870 grown on steam-exploded and hydrolyzed CS (cardoon stalks, **a**, **b**), SD1, SD2 (stranded driftwood, **c**, **d**), OTPR1 and OTPR2 (olive tree pruning residues, **e**, **f**) at 20 and 25 °C. C16:0 palmitic acid (hexadecanoic acid), C18:0 stearic acid (octadecanoic acid), Δ9C18:1 oleic acid [(9E9Z)-octadec-9-enoic acid], ∆9,12C18:2 linoleic acid [(9Z,12Z)-9,12-octadecadienoic acid]. SFA = % of saturated FA; UFA = % of unsaturated FA; UI = unsaturation index. **a**, **b** Na 20 and Na 25, and St 20 and St 25 = *N. adeliensis* and *S. terricola*, respectively, grown on CS at 20 and 25 °C. **c**, **d** Lc 20-1 and Lc 20-2 = *L. creatinivorum* grown on SD1 and SD2, respectively, incubated at 20 °C; Na 20-1, Na 20-2, Na 25-1 and Na 25-2 = *N. adeliensis* grown on SD1 and SD2, respectively, incubated at 20 and 25 °C; St 20-2 and St 25-2 = *S. terricola* grown on SD2, incubated at 20 and 25 °C. **e**, **f** Lc 20-1 and Lc 25-1 = *L. creatinivorum* grown on OTPR1, incubated at 20 and 25 °C; Na 20-1, Na 20-2, Na 25-1 and Na 25-2 = *N. adeliensis* grown on OTPR1 and OTPR2, respectively, incubated at 20 and 25 °C; St 20-1 = *S. terricola* grown on OTPR1, incubated at 20 °C

PCA was used to ordinate *L. creatinivorum*, *N. adeliensis* and *S. terricola* according to their FA profiles, % of SFA and UFA, and UI (Fig. 2). Interestingly, the sum of PC1 and PC2 was always close to 100% of total variance, thus suggesting that the experimental variables (different substrates and incubation temperatures) led to different patterns of lipid accumulation by the three oleaginous yeasts. Besides, in all cases, the discrimination among the strains, substrates and incubation temperatures was almost exclusively due to PC1 (from 89 to 99%) (Fig. 2).

The strains grown on CS showed no clustering tendency. *N. adeliensis* grown at both 20 and 25 °C exhibited a higher concentration of oleic acid, % of UFA and UI, differently from *S. terricola* grown at both 20 and 25 °C, which exhibited lower UI, due to its higher content of stearic and palmitic acid (Fig. 2a, b). Figure 2c, d report strains grown on SD1 and SD2. Only one cluster (including *L. creatinivorum* grown on both substrates at 20 °C and *N. adeliensis* grown on SD1 at 25 °C) was found. Also in this case, the concentration of oleic, stearic and palmitic acids, and the % of UFA and UI discriminated among different strains and growth temperatures (Fig. 2c, d). The PCA of strains grown on OTPR1 and OTPR2 allowed identifying only the cluster containing *N. adeliensis* grown on both substrates at 20 °C (Fig. 2e). Also on OTPR the different concentrations of oleic, stearic and palmitic acids, and the  % of UFA and UI discriminated among strains (Fig. 2e, f).

### Comparison of FA profiles of lipids produced by oleaginous yeasts with those obtained from some oilseed crops

**Fig. 3:**
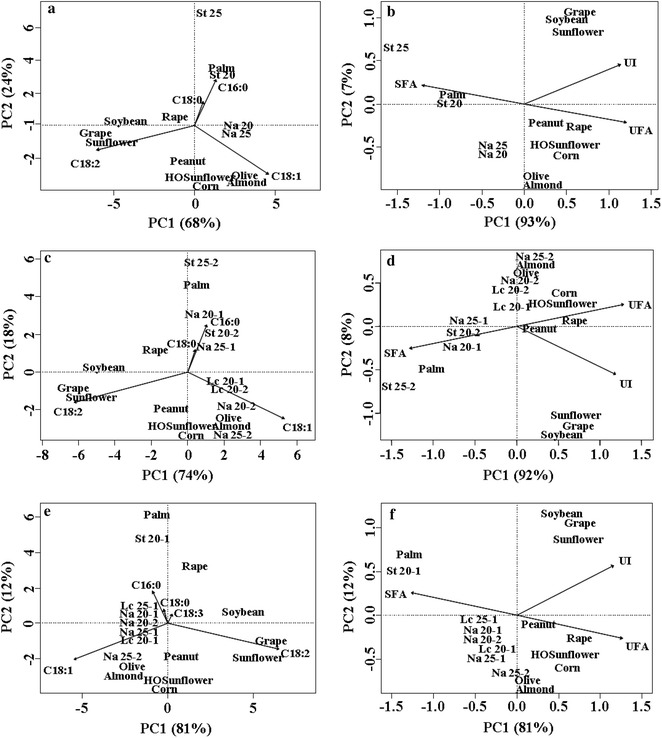
PCA of the composition of lipids produced by *Leucosporidium creatinivorum* DBVPG 4794, *Naganishia adeliensis* DBVPG 5195 and *Solicoccozyma terricola* DBVPG 5870 grown on steam-exploded and hydrolyzed CS (cardoon stalks, **a**, **b**), SD1, SD2 (stranded driftwood, **c**, **d**), OTPR1 and OTPR2 (olive tree pruning residues, **e**, **f**) at 20 and 25 °C and the composition of oils obtained from palm, olive, peanut, rape, soybean, sunflower, grape, H.O. sunflower, almond and corn oil. C16:0 palmitic acid (hexadecanoic acid), C18:0 stearic acid (octadecanoic acid), Δ9C18:1 oleic acid [(9E9Z)-octadec-9-enoic acid], ∆9,12C18:2 linoleic acid [(9Z,12Z)-9,12-octadecadienoic acid], ∆9,12,15C18:3 α-linolenic acid [(9Z,12Z,15Z)-9,12,15-octadecatrienoic acid]. SFA =  % of saturated FA; UFA =  % of unsaturated FA; UI = unsaturation index. **a**, **b** Na 20 and Na 25, St 20 and St 25 = *N. adeliensis* and *S. terricola*, respectively, grown on CS at 20 and 25 °C. **c**, **d** Lc 20-1 and Lc 20-2 = *L. creatinivorum* grown on SD1 and SD2, respectively, incubated at 20 °C; Na 20-1, Na 20-2, Na 25-1 and Na 25-2 = *N. adeliensis* grown on SD1 and SD2, respectively, incubated at 20 and 25 °C; St 20-2 and St 25-2 = *S. terricola* grown on SD2, incubated at 20 and 25 °C. **e**, **f**: Lc 20-1 and Lc 25-1 = *L. creatinivorum* grown on OTPR1, incubated at 20 and 25 °C; Na 20-1, Na 20-2, Na 25-1 and Na 25-2 = *N. adeliensis* grown on OTPR1 and OTPR2, respectively, incubated at 20 and 25 °C; St 20-1 = *S. terricola* grown on OTPR1, incubated at 20 °C

PCA was performed to compare the composition of lipids produced by *L. creatinivorum*, *N. adeliensis* and *S. terricola* grown on steam-exploded and hydrolyzed CS, SD1 and SD2, and OTPR1 and OTPR2 at 20 and 25 °C (Table 3) and those of oils obtained from some oilseed crops (i.e., palm, olive, peanut, rape, soybean, sunflower, grape, H.O. sunflower, almond, and corn oil), as reported by Ramos et al. [54]. Results are shown in Fig. 3.

Interestingly, the composition of lipids produced by *S. terricola* grown at 20 °C on CS overlapped almost perfectly with that of palm oil (Fig. 3a, b). On the other hand, lipids produced by *N. adeliensis* grown on SD2 at 20 °C and 25 °C and on OTPR2 at 25 °C exhibited a composition close to that of olive and almond oils (Fig. 3c–f).

In light of the above results, the possibility of using lipids produced by oleaginous yeasts from pre-treated lignocellulosic biomass feedstocks as sustainable and cheap extra source of oils exhibiting a lipid profile comparable with that of some oilseed crops (in particular palm, olive and almond) could appear a realistic chance, also in view of the rising consumer request of oleochemicals, which could determine a reduction of their availability and, consequently, a considerable increase of their price in industrial market [55, 56]. In this context, the possible supplementary use of yeast lipids could be regarded as a possible way to mitigate the problems associated with the cultivation of some oilseed crops [57, 58]. Accordingly, Whiffin et al. [52], reported that the environmental benefits of using lipids produced by oleaginous microorganisms including yeasts maybe considered significant.

### Predictive estimation of the physical characteristics of biodiesel potentially obtainable from lipids produced by oleaginous yeasts

Empirical formulas [59] were used for predicting the possible physical characteristics (according to European Standards EN 14214) of the biodiesel obtainable from lipids produced by *L. creatinivorum*, *N. adeliensis* and *S. terricola*. On the basis of above formulas high percentages of SFAs are positively correlated to the shelf—life of biodiesel in terms of oxidative stability (OS) and the combustion quality in terms of cetane number (CN), whereas high percentages of UFAs exhibit a positive correlation to cold flow plugging properties (CFPP) [59]. Fatty acid profiles (reported in Table 3), chain lengths and the number of double bonds were used as independent variables. The values are reported in Table 4.

**Table 4:** Predictive estimation of the physical characteristics of biodiesel potentially obtainable from lipids produced by *Leucosporidium creatinivorum* DBVPG 4794, *Naganishia adeliensis* DBVPG 5195 and *Solicoccozyma terricola* DBVPG 5870 grown on steam-exploded and hydrolyzed CS (cardoon stalks), SD1, SD2 (stranded driftwood), OTPR1 and OTPR2 (olive tree pruning residues) at 20 and 25 °C

Reference values/ranges^a^	T (°C)	OS (h]	CFPP (°C)	KV (mm^2^/s)	D (Kg/m^3^)	SV (mg)	IV (mg)	CN	HHV (MJ/Kg)
		6 h min	variable	3.5–5	860–900	0.50 min	120 max	51 min	not specified
CS									
*Naganishia adeliensis* DBVPG 5195	20	25.14	7.34	4.08	872.71	202.32	67.04	56.18	40.13
	25	23.43	12.07	4.13	872.67	201.47	67.35	56.22	40.16
*Solicoccozyma terricola* DBVPG 5870	20	27.11	15.93	4.11	871.12	204.49	52.86	59.51	40.25
	25	24.51	39.72	4.19	869.01	205.72	36.61	63.50	40.45
SD1									
* Leucosporidium creatinivorum* DBVPG 4794	20	13.53	− 4.07	4.00	874.68	201.83	81.91	52.45	39.93
* Naganishia adeliensis* DBVPG 5195	20	15.45	14.08	4.05	872.42	203.77	63.29	56.95	40.13
	25	16.27	4.60	4.02	873.23	203.24	69.19	55.51	40.06
SD2									
*Leucosporidium creatinivorum* DBVPG 4794	20	16.99	− 5.03	3.98	874.27	202.68	77.57	53.45	39.96
*Naganishia adeliensis* DBVPG 5195	20	26.96	1.01	4.09	872.87	200.50	75.88	54.17	40.07
	25	20.32	0.67	4.08	874.54	199.78	82.10	52.68	40.01
*Solicoccozyma terricola* DBVPG 5870	20	34.99	10.90	4.15	871.67	202.46	60.31	57.88	40.22
	25	35.26	34.65	4.18	869.07	204.21	43.85	61.85	40.40
OTPR1									
*Leucosporidium creatinivorum* DBVPG 4794	20	20.43	− 3.22	4.00	874.44	202.28	79.12	53.11	39.95
	25	19.08	4.14	4.02	873.22	203.34	71.03	55.03	40.03
*Naganishia adeliensis* DBVPG 5195	20	20.14	3.75	4.04	873.53	202.55	72.59	54.74	40.04
	25	30.14	1.47	4.06	873.48	202.26	72.35	54.84	40.05
*Solicoccozyma terricola* DBVPG 5870	20	38.22	23.72	4.14	871.17	203.65	53.82	59.38	40.27
OTPR2									
*Naganishia adeliensis* DBVPG 5195	20	23.17	1.29	4.03	873.44	202.88	72.32	54.76	40.03
	25	43.97	− 3.34	4.05	873.78	201.85	74.91	54.24	40.03

*OS* oxidative stability, *CFPP* cold filter plugging point, *KV* kinematic viscosity, *D* density, *SV* saponification value, *IV* iodine value, *CN* cetane number, *HHV* high heating value. OS, CFPP, KV, D, SV, IV, CN and HHV have been calculated as reported by Patel et al. [59]

^a^Selected technical specifications for biodiesel standards EN 14214 [59]

Overall, the predictive estimation of the physical characteristics of biodiesel potentially obtainable from lipids produced by *L. creatinivorum*, *N. adeliensis* and *S. terricola* gave results almost perfectly overlapping with reference values/ranges suggested by the European Standards EN 14214 (Table 4). Therefore, they could be taken into consideration as possible candidates for a supplementary production of a biodiesel with good performances [54, 59].

## Conclusions

Some Mediterranean biomass feedstocks may be used (after steam-explosion and hydrolysis) as carbon sources for lipid production by basidiomycetous yeasts. *S. terricola* DBVPG 5870 exhibited the highest lipogenic performances: its lipid composition after growth on CS at 20 °C was close to that of palm oil, while lipids produced by *N. adeliensis* DBVPG 5195 grown on SD2 and on OTPR2 at 25 °C showed a composition similar to those of olive and almond oils. Accordingly, yeast lipids herein studied could be used as extra sources of oils for producing biofuels and biochemicals. Further studies are in progress for a deeper characterization of the most versatile strain *N. adeliensis* DBVPG 5195, as well as the most productive one *S. terricola* DBVPG 5870.

## Abbreviations

FA: fatty acid
GC-MS: gas chromatography–mass spectrometry
TAGs: triacylglycerols
C/N: carbon/nitrogen
CS: cardoon stalks
SD: stranded driftwood residues
OTPR: olive tree pruning residues
WIS: water insoluble substrate
PL: pre-treatment liquor
S/L: solid/liquid
NREL: National Renewable Energy Laboratory
AIR: acid-insoluble residue
AIL: acid-insoluble lignin
HPLC: high performance liquid chromatography
RI: refractive index
A600: absorbance at 600 nm
C_Hy %_: enzymatic hydrolysis yields
*r*_Gc_: glucose anhydrous correction
f_G_: grams of glucose mass fraction
WIS_l_: grams of water insoluble substrate loaded into the bioreactor
C _%_: cellulose percentage in the WIS
PCA: principal components analysis
ANOVA: analysis of variance
SFA: saturated fatty acid
UFA: unsaturated fatty acid
UI: unsaturation index
P_L_: total lipid production
DW: dry weight
P_L_/DW: % of total intracellular lipid on cell biomass
P_L_/C: lipid yield
P_L_/d: lipid production per day
C6:0: caproic acid (hexanoic acid)
C8:0: caprylic acid (octanoic acid)
C10:0: capric acid (decanoic acid)
C12:0: lauric acid (dodecanoic acid)
C14:0: myristic acid (tetradecanoic acid)
C16:0: palmitic acid (hexadecanoic acid)
∆9C16:1: palmitoleic acid [(9Z)-hexadec-9-enoic acid]
C18:0: stearic acid (octadecanoic acid)
∆9C18:1: oleic acid [(9E9Z)-octadec-9-enoic acid]
∆9,12C18:2: linoleic acid [(9Z,12Z)-9,12-octadecadienoic acid]
∆9,12,15C18:3: α-linolenic acid [(9Z,12Z,15Z)-9,12,15-octadecatrienoic acid]
∆6,9,12C18:3: γ-linolenic acid [(6Z,9Z,12Z)-6,9,12-octadecatrienoic acid]
C20:0: arachic acid (eicosanoic acid)
∆11C20:1: gondoic acid [(11Z)-11-eicosenoic acid]
C22:0: behenic acid (docosanoic acid)
∆13C22:1: erucic acid [(13Z)-docos-13-enoic acid]
C24:0: lignoceric acid (tetracosanoic acid)
OS: oxidative stability
CFPP: cold filter plugging point
KV: kinematic viscosity
D: density
SV: saponification value
IV: iodine value
CN: cetane number
HHV: high heating value
